# Supplementary material for: Lung Protection Strategies during Cardiopulmonary Bypass Affect the Composition of Blood Electrolytes and Metabolites—A Randomized Controlled Trial
Source: J Clin Med. 2018 Nov 21;7(11):462. doi: 10.3390/jcm7110462 (PMC6262287; doi:10.3390/jcm7110462)
Supplement: Supplementary file 1 [file jcm-07-00462-s001.pdf]

Table S1. Overview of metabolite changes with time in each corresponding group

Metabolites		Pre-CPB (mM)			End CPB (mM)			2 Hours Post-CPB (mM)			4 Hours Post-CPB (mM)			6 Hours Post-CPB (mM)			20 Hours Post-CPB (mM)			Time	Time * Group
		Standard	HTK	O2	Standard	HTK	O2	Standard	HTK	O2	Standard	HTK	O2	Standard	HTK	O2	Standard	HTK	O2	*p*-value	*p*-value
Trigonelline #	Mean	0.004	0.007	0.007	0.005	0.009	0.006	0.004	0.009	0.005	0.004	0.006	0.006	0.004	0.006	0.005	0.003	0.003	0.003	0.002	0.01
	SD	0.003	0.009	0.007	0.005	0.008	0.006	0.004	0.009	0.005	0.004	0.006	0.005	0.004	0.006	0.004	0.003	0.003	0.004		
Formate #	Mean	0.012	0.011	0.012	0.012	0.013	0.012	0.011	0.012	0.011	0.011	0.012	0.010	0.010	0.011	0.010	0.010	0.011	0.010	2.35×10^{6}	0.01
	SD	0.003	0.001	0.002	0.003	0.002	0.002	0.003	0.002	0.002	0.003	0.002	0.002	0.002	0.002	0.002	0.002	0.002	0.002		
Phenylalanine #	Mean	0.055	0.055	0.052	0.071	0.072	0.073	0.064	0.074	0.067	0.061	0.070	0.060	0.056	0.068	0.060	0.073	0.087	0.077	2.61×10^{18}	0.02
	SD	0.012	0.009	0.012	0.017	0.016	0.020	0.018	0.016	0.018	0.017	0.022	0.017	0.015	0.022	0.016	0.016	0.021	0.016		
Tyrosine	Mean	0.038	0.037	0.036	0.043	0.041	0.043	0.039	0.034	0.039	0.039	0.035	0.036	0.034	0.033	0.033	0.038	0.043	0.043	1.90×10^{6}	0.67
	SD	0.009	0.010	0.010	0.012	0.011	0.012	0.010	0.009	0.010	0.013	0.010	0.010	0.009	0.011	0.010	0.011	0.013	0.010		
Glucuronate	Mean	0.002	0.004	0.010	0.004	0.005	0.012	0.037	0.054	0.052	0.054	0.062	0.061	0.064	0.069	0.064	0.060	0.040	0.049	4.72×10^{42}	0.39
	SD	0.00	0.01	0.02	0.01	0.01	0.02	0.02	0.04	0.03	0.02	0.04	0.03	0.03	0.04	0.04	0.05	0.04	0.06		
Histidine #	Mean	0.068	0.066	0.065	0.071	6.362	0.068	0.069	3.984	0.067	0.064	2.790	0.063	0.064	2.040	0.062	0.061	0.474	0.063	1.74×10^{82}	1.57×10^{187}
	SD	0.009	0.014	0.011	0.013	2.011	0.014	0.012	1.284	0.014	0.016	1.018	0.015	0.019	0.729	0.013	0.013	0.463	0.011		
Urea #	Mean	0.103	0.131	0.139	0.121	0.138	0.143	0.119	0.142	0.140	0.122	0.156	0.143	0.123	0.166	0.145	0.129	0.181	0.131	0.31	0.01
	SD	0.03	0.08	0.08	0.05	0.08	0.09	0.05	0.07	0.07	0.06	0.07	0.08	0.05	0.07	0.07	0.05	0.08	0.04		
Triacylglycine (TAG)	Mean	0.261	0.307	0.296	0.002	0.002	0.020	0.076	0.076	0.096	0.099	0.080	0.155	0.091	0.078	0.164	0.108	0.138	0.209	6.27×10^{24}	0.27
	SD	0.15	0.25	0.19	0.00	0.00	0.08	0.08	0.10	0.12	0.12	0.10	0.22	0.11	0.12	0.27	0.13	0.13	0.30		
Mannose	Mean	0.046	0.045	0.043	0.049	0.044	0.049	0.056	0.048	0.053	0.060	0.055	0.057	0.063	0.062	0.060	0.074	0.079	0.074	1.45×10^{32}	0.72
	SD	0.01	0.02	0.01	0.01	0.01	0.01	0.02	0.02	0.01	0.02	0.02	0.01	0.02	0.01	0.02	0.02	0.02	0.02		
Phosphoenol-pyruvate	Mean	0.011	0.011	0.010	0.013	0.015	0.013	0.013	0.014	0.014	0.014	0.014	0.014	0.014	0.013	0.013	0.011	0.012	0.012	2.32×10^{6}	0.93
	SD	0.004	0.004	0.004	0.004	0.004	0.004	0.005	0.005	0.004	0.006	0.004	0.005	0.005	0.005	0.005	0.005	0.005	0.005		
Myo-inositol	Mean	0.020	0.023	0.025	0.022	0.023	0.026	0.025	0.032	0.033	0.029	0.042	0.039	0.036	0.051	0.040	0.065	0.061	0.074	2.48×10^{17}	0.79
	SD	0.02	0.03	0.02	0.02	0.02	0.02	0.03	0.03	0.03	0.03	0.04	0.03	0.04	0.04	0.03	0.05	0.05	0.04		
Mannitol #	Mean	0.052	0.146	0.358	7.081	8.292	6.234	4.924	5.940	4.967	3.759	4.351	3.474	3.137	3.464	2.871	1.003	0.819	0.820	3.70×10^{128}	0.00016
	SD	0.02	0.44	1.31	2.14	2.48	2.37	1.33	1.95	1.58	1.37	1.64	1.10	1.34	1.38	1.15	0.92	0.89	0.66		
Creatinine	Mean	0.042	0.042	0.046	0.057	0.054	0.058	0.055	0.052	0.058	0.058	0.056	0.058	0.058	0.059	0.057	0.060	0.052	0.056	0.0007	0.99
	SD	0.01	0.02	0.02	0.02	0.02	0.02	0.02	0.02	0.03	0.02	0.02	0.03	0.03	0.02	0.02	0.03	0.03	0.02		
Creatine #	Mean	0.027	0.032	0.032	0.030	0.072	0.038	0.035	0.082	0.042	0.036	0.069	0.045	0.038	0.055	0.043	0.035	0.051	0.048	0.00003	3.53×10^{14}
	SD	0.01	0.02	0.02	0.02	0.03	0.02	0.02	0.04	0.03	0.02	0.04	0.03	0.03	0.03	0.03	0.02	0.03	0.04		
Lysine	Mean	0.133	0.135	0.132	0.173	0.190	0.161	0.162	0.167	0.153	0.141	0.125	0.126	0.120	0.103	0.110	0.098	0.101	0.111	3.82×10^{41}	0.07
	SD	0.05	0.04	0.04	0.03	0.04	0.04	0.03	0.04	0.04	0.03	0.04	0.03	0.02	0.04	0.03	0.03	0.03	0.03		
Citrate	Mean	0.041	0.043	0.045	0.071	0.064	0.073	0.067	0.063	0.071	0.062	0.063	0.064	0.061	0.057	0.061	0.049	0.051	0.053	2.22×10^{12}	0.97
	SD	0.01	0.01	0.01	0.02	0.01	0.02	0.04	0.03	0.03	0.02	0.04	0.03	0.02	0.03	0.02	0.02	0.01	0.03		
Acetoacetic acid	Mean	0.048	0.040	0.049	0.064	0.037	0.061	0.053	0.066	0.065	0.070	0.074	0.066	0.065	0.040	0.063	0.106	0.086	0.074	0.0001	0.48
	SD	0.05	0.02	0.03	0.08	0.04	0.04	0.04	0.05	0.05	0.06	0.06	0.07	0.05	0.04	0.08	0.08	0.07	0.07		
Acetone	Mean	0.013	0.011	0.013	0.022	0.017	0.020	0.029	0.024	0.026	0.033	0.029	0.029	0.032	0.028	0.030	0.025	0.027	0.022	9.14×10^{10}	0.98

	SD	0.02	0.01	0.01	0.02	0.01	0.01	0.03	0.01	0.01	0.03	0.02	0.02	0.02	0.02	0.02	0.02	0.02	0.02		
NAc-Glycoprotein	Mean	0.062	0.067	0.060	0.048	0.048	0.047	0.057	0.055	0.053	0.058	0.057	0.055	0.058	0.058	0.055	0.066	0.066	0.060	2.68×10^{18}	0.36
	SD	0.012	0.014	0.008	0.010	0.013	0.007	0.013	0.014	0.008	0.012	0.014	0.009	0.012	0.015	0.010	0.012	0.014	0.012		
Alanine #	Mean	0.224	0.230	0.222	0.339	0.618	0.334	0.285	0.565	0.306	0.273	0.493	0.302	0.264	0.462	0.306	0.213	0.288	0.256	4.73×10^{39}	4.66×10^{51}
	SD	0.06	0.07	0.07	0.08	0.13	0.10	0.08	0.14	0.10	0.10	0.14	0.10	0.10	0.14	0.11	0.06	0.11	0.11		
3-Hydroxybutyric acid (3-HBA)	Mean	0.071	0.090	0.074	0.180	0.101	0.181	0.122	0.146	0.179	0.157	0.130	0.142	0.138	0.088	0.156	0.172	0.166	0.155	0.008	0.63
	SD	0.08	0.08	0.06	0.24	0.16	0.14	0.14	0.17	0.16	0.14	0.11	0.17	0.13	0.11	0.22	0.16	0.14	0.16		
Isobutyric acid	Mean	0.159	0.141	0.201	0.161	0.150	0.228	0.125	0.142	0.190	0.091	0.107	0.141	0.072	0.086	0.122	0.018	0.013	0.022	1.41×10^{16}	0.14
	SD	0.15	0.11	0.15	0.15	0.13	0.20	0.11	0.13	0.21	0.08	0.09	0.15	0.06	0.08	0.14	0.04	0.02	0.02		
Monounsaturated fatty acid (MUFA) #	Mean	1.21	1.28	1.31	0.55	0.57	0.67	0.89	0.92	1.01	1.00	0.99	1.18	0.99	1.01	1.23	0.90	1.07	1.21	1.41×10^{23}	0.04
	SD	0.34	0.39	0.45	0.12	0.15	0.30	0.22	0.32	0.28	0.26	0.26	0.54	0.25	0.27	0.72	0.29	0.33	0.77		
Glycine #	Mean	0.186	0.177	0.182	0.223	0.261	0.202	0.173	0.273	0.166	0.135	0.232	0.137	0.129	0.216	0.130	0.128	0.157	0.125	1.89×10^{32}	5.92×10^{32}
	SD	0.05	0.03	0.05	0.05	0.05	0.06	0.04	0.05	0.05	0.04	0.05	0.05	0.05	0.05	0.05	0.04	0.05	0.04		
Glutamine #	Mean	0.485	0.452	0.451	0.492	0.743	0.452	0.436	0.672	0.412	0.382	0.551	0.362	0.357	0.482	0.364	0.425	0.479	0.418	5.03×10^{28}	3.28×10^{52}
	SD	0.05	0.06	0.06	0.08	0.12	0.08	0.07	0.13	0.08	0.08	0.11	0.08	0.08	0.09	0.08	0.10	0.11	0.11		
Glutamate #	Mean	0.115	0.120	0.118	0.130	0.168	0.129	0.111	0.153	0.109	0.092	0.123	0.089	0.082	0.104	0.085	0.084	0.096	0.085	2.61×10^{51}	3.83×10^{22}
	SD	0.03	0.03	0.03	0.02	0.03	0.03	0.02	0.03	0.02	0.02	0.03	0.02	0.02	0.02	0.02	0.02	0.02	0.02		
Arginine & Proline	Mean	0.035	0.031	0.031	0.046	0.051	0.044	0.042	0.044	0.040	0.038	0.035	0.036	0.033	0.030	0.034	0.029	0.031	0.034	1.10×10^{23}	0.25
	SD	0.011	0.010	0.012	0.011	0.012	0.013	0.010	0.013	0.011	0.010	0.010	0.011	0.009	0.010	0.011	0.011	0.009	0.008		
Isoleucine #	Mean	0.030	0.030	0.032	0.041	0.034	0.041	0.037	0.026	0.037	0.034	0.021	0.029	0.027	0.017	0.025	0.030	0.031	0.031	2.53×10^{12}	0.00006
	SD	0.008	0.007	0.010	0.013	0.012	0.015	0.013	0.013	0.017	0.014	0.011	0.016	0.011	0.010	0.015	0.011	0.008	0.011		
Valine	Mean	0.152	0.157	0.166	0.171	0.171	0.182	0.178	0.174	0.187	0.184	0.175	0.178	0.160	0.166	0.169	0.135	0.153	0.151	2.13×10^{7}	0.73
	SD	0.03	0.03	0.04	0.04	0.04	0.05	0.04	0.04	0.05	0.04	0.04	0.05	0.04	0.04	0.05	0.04	0.03	0.04		
Leucine	Mean	0.087	0.089	0.092	0.110	0.103	0.119	0.110	0.104	0.113	0.105	0.094	0.095	0.085	0.083	0.086	0.088	0.095	0.095	7.39×10^{7}	0.88
	SD	0.02	0.02	0.03	0.03	0.03	0.04	0.03	0.03	0.04	0.04	0.03	0.04	0.03	0.04	0.05	0.02	0.03	0.03		
Lipoproteins #	Mean	1.45	1.54	1.54	0.75	0.77	0.89	1.14	1.09	1.25	1.22	1.20	1.36	1.23	1.20	1.37	1.16	1.36	1.40	5.44×10^{39}	0.009
	SD	0.33	0.29	0.41	0.14	0.14	0.28	0.25	0.24	0.32	0.23	0.20	0.40	0.24	0.25	0.46	0.25	0.28	0.47		
Acetate	Mean	0.110	0.102	0.095	0.111	0.097	0.103	0.086	0.083	0.081	0.079	0.067	0.070	0.065	0.052	0.061	0.041	0.037	0.039	2.66×10^{65}	0.11
	SD	0.045	0.029	0.036	0.033	0.018	0.027	0.011	0.021	0.017	0.016	0.021	0.017	0.016	0.017	0.018	0.018	0.013	0.019		
Arginine & Citrulline	Mean	0.100	0.082	0.095	0.156	0.166	0.152	0.146	0.144	0.135	0.127	0.104	0.111	0.095	0.078	0.101	0.085	0.081	0.097	1.69×10^{30}	0.34
	SD	0.04	0.04	0.05	0.04	0.04	0.06	0.04	0.05	0.05	0.04	0.04	0.04	0.04	0.04	0.04	0.04	0.04	0.04		
Methionine	Mean	0.011	0.008	0.011	0.015	0.013	0.014	0.011	0.012	0.012	0.008	0.010	0.009	0.006	0.009	0.009	0.010	0.011	0.012	2.75×10^{6}	0.75
	SD	0.008	0.005	0.007	0.009	0.009	0.008	0.008	0.008	0.007	0.006	0.008	0.007	0.005	0.007	0.007	0.008	0.008	0.008		
Threonine #	Mean	0.093	0.089	0.086	0.097	0.123	0.088	0.082	0.120	0.077	0.074	0.109	0.069	0.066	0.102	0.067	0.069	0.092	0.069	4.21×10^{14}	1.01×10^{28}
	SD	0.02	0.02	0.03	0.02	0.03	0.02	0.02	0.03	0.02	0.02	0.02	0.02	0.02	0.03	0.02	0.02	0.03	0.02		
Aspartate #	Mean	0.099	0.088	0.093	0.091	0.121	0.085	0.081	0.116	0.074	0.074	0.105	0.072	0.065	0.094	0.061	0.069	0.090	0.070	9.53×10^{8}	4.28×10^{13}
	SD	0.006	0.006	0.006	0.006	0.006	0.006	0.006	0.006	0.006	0.006	0.006	0.006	0.006	0.006	0.006	0.006	0.006	0.006		
Fucose	Mean	0.011	0.011	0.012	0.014	0.015	0.016	0.015	0.018	0.018	0.018	0.018	0.019	0.017	0.018	0.019	0.015	0.015	0.016	3.58×10^{16}	0.89
	SD	0.004	0.006	0.005	0.006	0.006	0.006	0.005	0.008	0.005	0.005	0.008	0.005	0.005	0.006	0.005	0.005	0.005	0.005		
Free Fatty acid (FFA) #	Mean	0.679	0.661	0.733	0.036	0.022	0.074	0.208	0.208	0.289	0.279	0.288	0.407	0.273	0.287	0.442	0.292	0.459	0.597	7.89×10^{31}	0.05
	SD	0.38	0.35	0.48	0.12	0.06	0.18	0.15	0.15	0.24	0.20	0.18	0.42	0.20	0.23	0.54	0.21	0.25	0.67		

Pyruvate #	Mean	0.021	0.024	0.023	0.049	0.070	0.055	0.036	0.047	0.046	0.043	0.055	0.059	0.050	0.064	0.063	0.032	0.042	0.051	5.08×10^{23}	0.0004
	SD	0.01	0.01	0.01	0.02	0.02	0.02	0.02	0.02	0.03	0.03	0.03	0.03	0.03	0.03	0.03	0.01	0.02	0.03		
Glucose	Mean	6.11	6.46	6.34	7.36	7.65	7.91	7.69	8.65	8.30	8.75	9.40	9.15	9.06	9.28	8.85	7.72	8.01	7.90	3.60×10^{23}	0.75
	SD	0.98	1.44	1.31	1.82	1.53	1.58	1.57	2.69	1.60	2.07	2.55	1.74	1.58	2.31	1.59	1.97	1.40	1.42		
Lactate	Mean	0.69	0.72	0.78	1.66	1.94	1.89	1.24	1.50	1.49	1.57	1.87	1.87	1.61	2.22	2.21	1.37	1.31	1.97	5.28×10^{10}	0.43
	SD	0.23	0.22	0.31	0.52	0.62	0.65	0.64	0.84	0.54	1.05	1.62	0.91	0.99	1.76	1.64	1.17	0.58	3.19		
2-Ketoglutarate #	Mean	0.048	0.047	0.046	0.045	0.044	0.042	0.041	0.043	0.040	0.041	0.043	0.040	0.038	0.041	0.039	0.038	0.039	0.036	0.0002	0.05
	SD	0.003	0.003	0.003	0.002	0.003	0.003	0.003	0.003	0.003	0.002	0.003	0.003	0.003	0.003	0.003	0.003	0.003	0.003		

Two-way ANOVA and its corresponding Tukey's post-hoc test for group comparison were used to detect differences between patients as a consequence of surgery (time) and treatment received (time-group interaction). A two-tailed *p*-value ≤0.05 was considered significant. Metabolites marked (#) were found to be significant for both time, and the time–treatment interaction.
